# Genotyping *Rickettsia prowazekii* Isolates

**DOI:** 10.3201/eid1408.080444

**Published:** 2008-08

**Authors:** Yong Zhu, Aaron Medina-Sanchez, Donald Bouyer, David H. Walker, Xue-jie Yu

**Affiliations:** * University of Texas Medical Branch, Galveston, Texas, USA

**Keywords:** *Rickettsia prowazekii*, genotype, epidemic typhus, dispatch

## Abstract

We developed a typing method that can differentiate 8 strains of *Rickettsia prowazekii* into 7 genotypes. This method can be used to type and trace the origin of *R. prowazekii* isolated from samples collected during epidemics after a bioterrorism attack.

*Rickettsia prowazekii* is the causative agent of epidemic typhus and also a potential bioterrorism agent. The disease may occur in epidemics when social, economic, or political systems are disrupted and expose a large population such as refugees to louse infestation due to lack of hygiene. Recent outbreaks of typhus have occurred in Burundi, Algeria, Peru, and Russia (*1*,*2*). *R. prowazekii* is transmitted by the human body louse, *Pediculus humanus corporis,* in the human cycle. Sylvatic typhus associated with *R. prowazekii* has been documented in the eastern United States. However, it is not clear whether *R. prowazekii* transmission to humans from flying squirrels results from the bite of fleas or lice or contaminated arthropod fecal material (*3*,*4*). Reemergence of epidemic typhus and the potential use of *R. prowazekii* in bioterrorist attacks requires a molecular method that can type isolates and trace the origin or epidemiology of the disease.

## The Study

Our objective was to identify a minimal gene set in which PCR amplification and sequencing would allow the efficient differentiation of *R. prowazekii* strains for diagnostic purposes. Using BLAST analysis (www.ncbi.plm.nih.gov/blast/b12seq/wblast2.cgi) to identify target DNA sequences for genotyping, we compared the genomic sequences of Madrid E strain (E strain, NC_000963) (*5*) with those of Nuevo Leon strain, a new tick isolate of *R. prowazekii* (*6*), which was sequenced recently (unpub. data). We identified 6 loci with insertion or deletion in 1 of 2 strains. PCR primers were designed from the target sequences and used to amplify DNA from 8 strains of *R. prowazekii*, including human isolates Addis Ababa, Breinl, Cairo, and E strain; a guinea pig isolate of Evir strain (*7*); a tick isolate (ZRS) from Ethiopia (*8*); and 2 flying squirrel isolates (GvV-250 from Virginia and GvF-16 from Florida) (Table 1) (*4*). Rickettsial genomic DNA was extracted from the *R. prowazekii–*infected L929 cells or infected yolk sacs of embryonated chicken eggs by using the GenElute Mammalian Genomic DNA Miniprep kit (Sigma-Aldrich, St. Louis, MO, USA) according to the manufacturer's instructions.

**Table 1:** Primers for 6 loci of *Rickettsia prowazekii* genomic DNA sequences

Target sequence	Sequences of forward primer/reverse primer (5′ → 3′)	PCR product size, bp	Reference
*rp028*	TTGATATAGGTTGCGGAGTCGGTGTTA/TCATTGATGGCTTGTAGTTTTTCTGCT	682	(*9*)
*rp181*	ATTATGCAAATAATGCAG/GCATCGGATAAGTTAGTTCA	390	This study
*rp195*	TTTATTGGGGATTTACCTTT/CAAGTGTTAGATAGCTTGCT	384	This study
*rp272/rp273*	TCTTGCGATACAGTAAGCAC/TATTCGCTCCTTACCAGTTA	612	This study
*rp308/rp309*	TTAACAGAAGTAATAATAATTG/AGCAATAGAATTTGATAAGCA	369	This study
*rp691/rp692*	AGAAATTTGTATTGCATTTTTATG/GCTCTAGAAGCTATTGCTGA	447	This study

For designing the primers (Table 1), we used Primer 3.0 software (http://frodo.wi.mit.edu/cgi-bin/primer3/primer3_www.cgi); primers were synthesized. Two microliters of the DNA preparation were amplified in a 50-µL RED taq ReadyMIX PCR reaction (Sigma-Aldrich). The following conditions were used for amplification: an initial 5 min of denaturation at 94°C followed by 30 cycles of denaturation for 30 s at 94°C, annealing for 30 s at 53 °C, and extension for 1 min at 72°C. Amplification was completed by holding the reaction mixture for 2 min at 72°C. PCR products were directly sequenced with PCR primers for both strands. PCR amplification and DNA sequencing were performed twice for each gene of each *R. prowazekii* strain. A PCR reaction without template DNA was included as a negative control in each PCR.

DNA sequences were aligned by using DNASTAR Lasergene software, version 6.0 **(**DNASTAR, Inc., Madison, WI, USA). The sequences amplified by 6 pairs of primers from each strain were joined together to form a concatenated sequence for each strain. A multiple alignment of the concatenated sequences was constructed by using ClustalW (www.ebi.ac.uk/clustalw) and was analyzed by using the neighbor-joining method in PAUP 4.0 Beta (Sinauer Associate, Inc., Sunderland, MA, USA). Bootstrap was estimated for neighbor-joining trees by 1,000 resamplings. The sequences reported here were assigned consecutive GenBank accession numbers from EU192931 to EU192949.

## Conclusions

We amplified the 6 loci from all 8 *R. prowazekii* strains and compared the corresponding sequences of each strain to identify the variations among strains. Three loci were intergenic spacers (*rp272/rp273*, *rp308/rp309*, and *rp691/rp692*), and 2 loci were pseudogenes (*rp181* and *rp195*) in all *R. prowazekii* strains. We also sequenced *rp028*, the methyltransferase gene, because we wanted to know if this gene was inactivated in any virulent strain of *R. prowazekii*. Pseudogene *rp028* was inactivated in a virulent E strain but not in its virulent revertant Evir strain (*9*). Coincident with inactivation of the methyltransferase gene, E strain is deficient in methylation of surface proteins (*10*,*11*).

Our result shows that a single nucleotide insertion at position 732 in *rp028* occurred only in E strain among the tested *R. prowazekii* strains (Table 2). However, single nucleotide polymorphism (SNP) existed in *rp028* among strains of *R. prowazekii* and was very useful in the differentiation of *R. prowazekii* strains (Table 2). Apparently none of these nucleotide substitutions caused attenuation of E strain because the E strain and Evir strain were identical at these sites.

**Table 2:** Genotypes of *Rickettsia prowazekii* strains determined by nucleotide mutation in multiple loci

Strain	rp028*					*rp181*		*rp195*			*rp272–rp273*		*rp308–rp309*		*rp691–rp692*		GT
	268†	286	480	732		713–714		140	1529		52–53		306		1306–1307	1415	
GvV-250	T	G	C	–		–		TACTTCAAGCTCATTTCG	C		AA		GTCATTATCGTAT		TT	G	1
GvF-16	T	G	T	–		–		TACTTCAAGCTCATTTCG	C		AA		GTCATTATCGTAT		TT	G	2
Breinl	T	A	C	–		–		TACTTCAAGCTCATTTCG	G		–		GTCATTATCGTAT		–	–	3
Cairo	T	A	C	–		G		TACTTCAAGCTCATTTCG	G		A		GTCATTATCGTAT		–	–	4
ZRS	G	A	C	–		G		–	G		AA		–		TT	–	5
Addis Ababa	G	A	C	–		G		–	G		AA		–		TT	–	5
Madrid E	G	A	C	A		GG		–	G		AA		–		TT	–	6
Evir	G	A	C	–		GG		–	G		AA		–		TT	–	7

*Gene names or intergenic spacers between genes. †Positions of nucleotides with mutation, which were counted from the first nucleotide of the coding sequence or the first nucleotide after the stop codon in the case of intergenic spacers; –, deletion of nucleotides, in which the number of nucleotides deleted equals the nucleotides in the same column for the corresponding strains that do not have the deletion. For example, in *rp181,* the GvV-250 strain has 1 deleted nucleotide when compared with the Cairo strain, but it has deleted 2 nucleotides when compared with the E strain.

DNA sequence comparison and phylogenetic analysis of the concatenated sequences indicated that the *R. prowazekii* strains were grouped together by geographic location and source of isolation (Table 2, Figure). Two flying squirrel isolates from the United States were differentiated by a single nucleotide substitution at position 480 in *rp028*. E strain and its revertant Evir strain differed by a single nucleotide insertion in E strain at position 732 in *rp028*, which we reported previously (*9*). Breinl and Cairo strains were closely related but were differentiated by several deletion/insertion mutations in *rp18*1 and the spacer between *rp272* and *rp273*. The cattle tick isolate ZRS and the human isolate Addis Ababa, both from Ethiopia, were identical in all 6 loci. ZRS strain and Addis Ababa strain were phylogenetically more closely related to E/Evir strains than other strains (Figure). There was only a single nucleotide difference between ZRS/Addis Ababa strains and Evir strain (Table 2).

**Figure:**
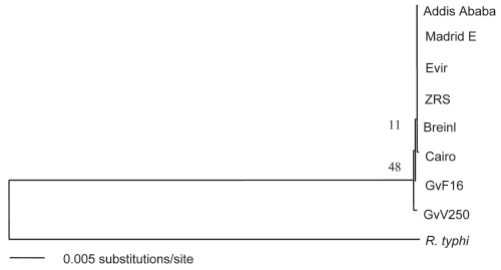
Phylogenic tree of *Rickettsia prowazekii* strains generated by using the concatenated sequences of 6 loci from each strain. *R. typhi* sequences were used to root the tree.

Genotyping of *R. prowazekii* has been explored recently. Zhu et al., using intergenic spacers *rpmE*/tRNAfMet and *serS/virB*4, differentiated 5 strains and PCR amplicons from 10 body lice of *R. prowazekii* into 4 genotypes (*12*). Ge et al. showed that *R. prowazekii* Breinl strain and E strain were different in the *rp084* gene, which was deleted from the Breinl strain (*13*). However, using the *rpmE*/*tRNAfMet* intergenic spacer, we were able to classify the 8 strains of *R. prowazekii* tested into only 2 genotypes. Genotype 1 contains Breinl strain and genotype 2 includes all other strains. All 8 strains were identical in the *serS/virB*4 spacer. With the exception of *R. prowazekii* Breinl strain, *rp084* was not deleted from any strains of *R. prowazekii* tested in our study. Conversely, using our methods, the 8 strains of *R. prowazekii* can be differentiated into 7 genotypes. ZRS and Addis Ababa strains are the only isolates that cannot be differentiated with our method. Because all *R. prowazekii* ZRS and Addis Ababa strains originated from Ethiopia, it is reasonable to believe that they might be genetically identical. Ge et al. recently showed that 5 *R. prowazekii* strains, including Breinl, Cairo, E, GvV257, and GvF12 were different from each other by 1 to 4 SNPs in *ompB* and *sca4*, respectively (*14*). However, the differentiation of *R. prowazekii* based on SNPs between closely related strains may be complicated by PCR and sequence errors. Conversely, our method confers more confidence in the validation of the mutations because we differentiated all strains except for 2 flying squirrel strains by insertion and deletion mutations, which are rarely generated by PCR or sequence errors.

Our method provides a technique for typing and tracing the origin of new *R. prowazekii* isolates. This method will have a broad use in the biodefense against and the molecular epidemiology of *R. prowazekii* and in detection of laboratory cross-contamination of *R. prowazekii* strains.
